# Exploring Health-Related Needs of Elderly People (70+) at Home: A Qualitative Study From Switzerland

**DOI:** 10.1177/21501327211055635

**Published:** 2021-11-23

**Authors:** Brunner Karma, Busch Ada-Katrin, Daniela Händler-Schuster

**Affiliations:** 1 Zurich University of Applied Sciences, Winterthur, Switzerland; 2 UMIT TIROL–Private University for Health Sciences, Medical Informatics and Technology, Hall in Tirol, Austria

**Keywords:** community care, healthy aging, elderly needs, specialized nurse

## Abstract

**Background:**

The elderly represents the fastest growing group in our population. Since there is a close relationship between the number of older people and health care expenditure, promoting healthy aging has become an important topic. However, it is essential to understand first the needs of this population in order to create suitable programs and activities.

**Methods:**

A qualitative design was used in this study to explore the subjective views of elderly people and to learn more about their health-related needs. A total of 12 participants were recruited using a consecutive sampling strategy. The data were collected through semi-structured interviews and analyzed by employing a summarizing content analysis.

**Results:**

This study has identified 4 areas of health-related needs: Independence and autonomy, social security, structure in daily life and community and belonging, where all categories are interrelated and interact with one other.

**Conclusion:**

Focusing more on community-based approaches would support creating a conducive environment. Also, home visits undertaken by a specialized nurse focused on health risks and disabilities could be an adequate approach to support the elderly population in an efficient way and to offer targeted programs and activities.

## Introduction

The elderly represents the fastest growing group in our global population.^1^ Between 2018 and 2050, the number of people aged over 64 is expected to increase by 70%,^2^ which raises the burden on the health care system^3^ as the need for healthcare services increases with age.^4^ In the last third of life, expenses are found to be the highest,^4^ which signifies a close relationship between the number of elderly people and health care expenditure.^1,5^ Also, the number of care places per capita in affluent countries such as Switzerland has been decreasing for several years.^6^ Consequently, the number of older people who need help at home will grow in the future. However, the current health care structure does not meet the needs of this population, as it has mainly focused on acute medical care so far.^4,7,8^

These sociodemographic developments reiterate the importance of healthy aging and raise the question of how to deal with critical issues such as frailty.^8^ Frailty and pre-frailty are a state of vulnerability due to various stressors that have a negative impact on a person’s health. Frailty, which means a reduced intrinsic capacity,^9^ can be used to represent the process between successful aging and experiencing disability.^8^ Previous research has identified different risk factors such as low physical activity, alcohol use, smoking, high body mass index, and unhealthy diet.^5^ Successful aging or the occurrence of frailty is often closely linked to the lifestyle of elderly people and possibly the need of behavioral changes.

A promising indicator on the successful implementation of behavioral changes in daily life is self-efficacy,^10^ which is based on the cognitive conviction that a problem can be solved by the individual’s effort. According to Bandura^10^ the self-efficacy perception has a direct effect on whether a problem is primarily accepted or not. Self-efficacy is affected by the environment in different ways,^10^ and previous research has pointed out the importance of the social and physical settings in which people conduct their daily activities^11^ as well. Therefore, how the community context affects health has been a research area of increasing interest.^12,13^

There are other health areas that have been more involved with the role of community. For instance, the *circle of care* theory by Abel,^14^ with its origins in end-of-life care, conceptualizes that an individual can be purposefully supported by strengthening their environment and community. This assumption is based on different dimensions: a person stands in the center and is surrounded by the different areas of their social environment. The dimensions closer to the person, such as the inner and outer network and the community, affect a person in an earlier stage and in a more direct way compared to service delivery or professional care.^14^

In Switzerland, there are healthcare providers to support the healthy and independent aging of this population with a plethora of services.^15^ One of these providers is Pro Senectute, the largest professional organization in Switzerland serving elderly people with a wide range of services, including leisure activities, nutrition, exercise, and other age-related activities. However, a large-scale study involving people aged over 64 years has shown that 38.5% in Switzerland were affected by pre-frailty.^16^ Therefore, to prevent or delay the need for elderly care, new approaches need to be developed to promote healthy aging and ensure adequate health care in the future.^6^ In order to establish health related solutions that support this population in a suitable way and strengthen their self-efficacy, it is necessary to first understand their point of views. However, such knowledge about the health-related experiences and needs that elderly persons have in their daily lives are lacking. Therefore, the aim of this study was to explore the health-related needs of elderly people (70+) living at home, and to derive practical suggestions on building a conducive environment, which can be implemented by service providers in Switzerland. Furthermore, the results of this study can provide useful insights to help establish a more community-centered approach to support elderly people.

## Methods

A qualitative exploratory design was used in this study to garner the subjective views of the elderly and to learn more about their needs in a health-related context to recommend suitable services. Participants were selected by consecutive sampling. The analysis was based on a qualitative text analysis, inductive approach, not only allowing the subject to be studied with the principle of openness without a predefined concept, but also enabled an in-depth exploration of respondents’ experiences.

### Participants

The recruitment strategy was based on the distribution of informative flyers by *Pro Senectute* in Winterthur, *seniors for seniors Winterthur*, as well as the church community Wülflingen. All participants were located in Winterthur, which unites urban and rural aspects and has about 114 200 residents. The inclusion criteria specified the minimum age of 70 years and the condition of living in their own homes. Interested potential participants were contacted by phone to answer some questions and to arrange for an appointment for the interview. Finally, 12 persons who fulfilled the inclusion criteria were selected for this study.

### Data Collection

Data were collected from 8 individual interviews and, at the request of the participants, 2 pair interviews. All Interviews were face-to-face and semi-structured in the interviewees mother tongue, Swiss German, and conducted between November 2020 and February 2021 by the first author. At the beginning of each interview, an information sheet was discussed and any questions were answered to ensure informed consent, followed by obtaining a declaration of permission, which could be withdrawn at any time. The interviews were audio-recorded and transcribed afterward verbatim in German. Additionally, field notes were taken by the researcher after each interview to support the analysis process later. All interviews were conducted in participants’ own homes, whenever possible, and lasted between 53 and 89 min. A declaration of non-competence was previously obtained from the ethics committee swissethics (Req-2020-00853).

### Data Analysis

After transcribing the anonymized interviews, the data were processed employing a summarizing content analysis, a systematic, rule guided qualitative text analysis method developed by Mayring.^17^ Performing the analytic 7-step process requires identifying a unit of analysis, followed by paraphrasing, generalizing, and reducing in order to condense the data systematically into the essential content. To reduce the data in an objective and sufficient way, this process was repeated several times. Using the data processing program MAXQDA version 2018.2, a code was created and assigned for each meaningful sentence, then similar codes were grouped into overarching subthemes, which were finally combined into main themes. During this inductive analysis, 4 main categories were established, representing the different needs expressed by the participants. The quotes that best fit into these categories are listed in each category to support the results.

## Results

**Figure 1.**
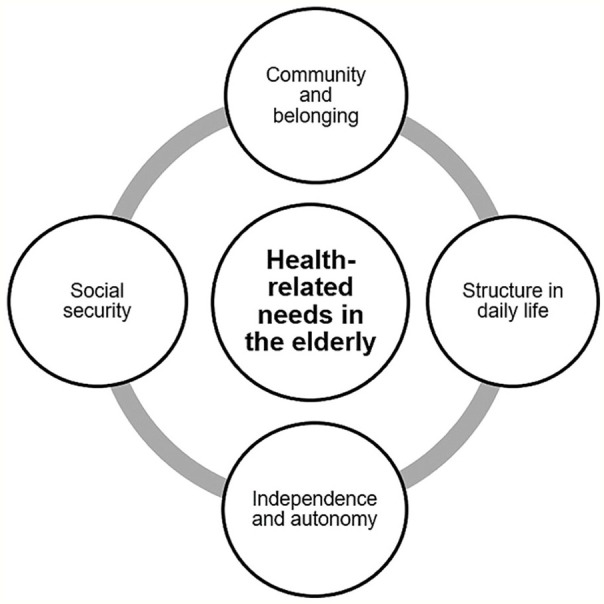
Results health-related needs in the elderly. Source: Own illustration.

All 12 interviewees participated in this study were retirees, the youngest was 70 and the oldest 89 years old. About 9 were female and 3 were male. The analysis identified 4 main categories, as illustrated in Figure 1: (1) Independence and autonomy, (2) social security, (3) structure in daily life, and (4) community and belonging. Within the categories community and belonging as well as social security, 2 sub-themes were formed: (a) continuous social contacts and (b) establishing a supportive network and getting assistance from the local community.

The health-related needs expressed mainly referred to the environmental conditions and influences faced by people during aging. The participants described which preparations they take to age healthily and which factors enhance this process or make it more difficult. The community was the dominant aspect and was mentioned in every Interview. All categories are interrelated and interact with each other.

### Independence and Autonomy

Independence and autonomy were frequently mentioned, along with the wish to stay in their own homes. It was not easy to accept help, especially from strangers, and some even considered this as shameful. Interviewees explained this as regressing from adult independence and they feel like a burden to others. The fear of losing personal autonomy in the future is a major concern, and 2 aspects, making independent decisions as well as implementing those decisions, were highlighted. This can be seen in the following quote made of 1 participant:

*“So no, I must be able to decide for myself and not have others make decisions for me. No, I don’t want that.” (Female, 74 years old).*

Staying independent and autonomous often requires building a supportive network and consulting trustworthy persons. Nevertheless, the idea of being at the mercy of someone or leaving home and losing the ability to decide for themselves caused great anxiety for many interviewees.

*This is reflected in the statement of this 75-year-old participant, who has a very active and independent lifestyle: “I am afraid of being helpless and at someone’s mercy.” (Female, 75 years old).*

Often, this worry is also closely associated with a potential diagnosis of dementia and the loss of cognitive abilities, as in this statement by a sprightly woman, who lives in a 2 rooms apartment:

*“Of course, everyone is afraid of that. That one day you might be dependent or have dementia. I’m afraid of that, too.” (Female, 89 years old).*

### Social Security

This category is divided into the sub-themes of financial security and the need for a person of trust. Both relate to social security, yet they are entirely different in their content.

Financial security is perceived as important. Accepting supplementary benefits by the state is seen as something dishonorable and it is perceived as unfair to have worked and still not be financially independent in old age. Nevertheless, the financial situation influences daily life and assistance for people with limited financial resources must be provided. These financial differences among retirees are reflected in this statement by a 71-year-old widower:

*“And then I realise that now I have time and I’m financially secure. (. . .) But it’s not the same for everyone. I also know people who are not doing so well and still have to earn something after they retire.” (Male, 71 years old)*

Furthermore, an even higher importance is attached to having a person of trust who is easy to reach if difficulties are encountered in daily life. The general practitioner is often mentioned in this context. People are willing to travel further distances to find a suitable trustworthy person like this 75-year-old women, who travels 40 min across town by bus to reach her general practitioner:

*“Yes, I have a long way to get to her, but I don’t mind. (. . .) She promised me that she would make home visits.” (Female, 75 years old).*

At the same time, home visits are mentioned as important and ensure a feeling of security.

The health behavior of the elderly is also closely linked to this person of trust. Feeling accepted and being able to speak openly are aspects that should be fulfilled in this regard. The importance of such an exchange is reflected in the statement of a participant, who changed her general practitioner several times until she finally felt comfortable:

*“She has time and she listens whenever I have something to say. I can talk and she has time. She never seems rushed that I think, oh, Important to avoid are therefore experiences of having not enough time, little humanity as well as inhibition barriers or the feeling of being compelled to follow instructions, such as taking medications.she must be stressed or something. No, she’s a really great one. And I can go to her anytime I have a problem.” (Female, 78 years old).*

There were also many bad experiences expressed exemplary described by this 84 year old married man

*“What I miss about the general practitioner is the personal touch. He types everything into his computer. He used to do more, examine me. The humane aspect is missing. (. . .) Overcoming an inhibition to go into something deeper and you don’t want to ask trivial things. Maybe I don’t have enough courage to bring things up.” (Male, 84 years old).*

### Structure in Daily Life

A structure in daily life is described as a supporting factor to keep activities and behavior in place, being health-promoting as well as maintaining social contacts, thus leading to a feeling of success. This statement of a participant reflects that it is important to plan activities that bring sense to everyday life:

*“And I personally need some kind of structure, too. I have to do something. I can’ t get up in the morning and think: what should I do now? I can’t always knit. And I cannot always read either. Right? And I mean, I can’t go for a walk all day either.” (Female, 78 years old).*

Sometimes, interviewees experienced difficulties to motivate themselves daily and to stick to plans. A structure in daily life such as regular times for eating and sleeping, or preparing a daily or weekly schedule is useful, as described by this participant who, with his wife, has established a regular daily structure to make it easier for him to get up in the mornings:

*“And we have a certain rhythm, always going to bed and getting up at the same time. And (. . .) we have breakfast at seven o’clock. Between quarter to twelve and twelve o’clock we have lunch, and at quarter to six we have dinner. And it’s always around the same time. And I think that’s a good thing.” (Male, 84 years old)*

### Community and Belonging

Two sub-themes have emerged to describe why a community is perceived as important to the participants.

First, finding like-minded people and experiencing the same continuous social contacts are perceived as important to acquire a sense of belonging. As the fear of loneliness has been described frequently, it is essential for the interviewees to feel connected, to interact, and have a reason to get up in the morning. The importance of this sense of belonging is expressed in this participant’s statement:

*“It gives me pleasure. We are a joyful group there. Also, there are always the same people at this time.” (Female, 78 years old)*

Second, establishing a supportive network and getting assistance from the local community is considered essential in healthy aging. Possible sources of help must be found in the close surroundings to ensure easy accessibility. Therefore, neighbors are an important resource for elderly people and the community is deemed crucial, especially if individuals become socially isolated because of worsening health or limited mobility. Consequently, it was noted that it is important to build a network before the need for help actually appears, like described by this 78-year-old female:

*“If you know, you could call there one day (. . .). This is important. And, as I said, you have to make friends. You cannot go looking for friends in a situation where you already need them. You have to find them before.” (Female, 78 years old).*

Close-knit neighborhoods, where everyone knows each other, are supportive environments. The participants experienced the risk of being alone with growing age and men are estimated to be more susceptible to this because they are perceived to be not as good as women in networking, hence requiring more support.

A commonality in both sub-themes is the realization that the maintenance of social contacts is not always an easy task—especially for persons with few social contacts or who have lost their spouse. Nevertheless, a proactive attitude toward this is apparent where participants try to change their own behavior and habits to become part of a community. One participant, for example, shared that for this purpose she is actively involved in the church community, even though she describes herself as non-religious:

*“Yes, let’s say (. . .) I have already tried to network somehow. For example, I help in the church (. . .). I already try to make my connections. Although I’m not insanely religious or anything like that. But the church does a lot for the elderly (. . .s).” (Female, 75 years old).*

It was mentioned several times that health-related activities are more likely to be attended regularly within a recurring group. The community is described as essential because family members often do not live in the same area.

## Discussion

To cope with future challenges with the growing number of elderly people and the increasing health costs, developing suitable services for healthy aging is essential. This research has explored the health-related needs of elderly people (70+) living at home. Through a qualitative approach based on semi-structured interviews and a summarizing content analysis, 4 main areas have been identified:

First, seeking independence and autonomy, as already emphasized in previous research,^18,19^ holds a key position in the lives of the population of interest. This might seem paradoxical as compared to other categories but previous research has shown that contextual conditions, social factors, as well as health-care professionals are important factors that highly affect physical function and therefore influence the level of independence and autonomy.^20,21^

Second, social security is essential—besides financial considerations the need for a person of trust was mentioned frequently. One participant brought up the idea of nurses visiting elderly people at home for preventive care. Having a specialized nurse visit older people in their homes could help identify potential risk factors like polymedication and discuss individual solutions. This practice could include an adapted form of frailty assessment, which is commonly used in acute geriatric patients.^9,22^ A study from Sweden showed that integrating such an assessment in the ambulant field can potentially engender high cost savings in the health-care sector,^23^ further recommending that a general practitioner undertakes this. This, however, contradicts the results of our study as the participants described it as difficult to find a suitably patient and caring general practitioner. Additionally, there were barriers to address certain issues and poor health behaviors, like bad compliance regarding medication management, were explained with lack of personal motivation because they felt compelled.

These sentiments support the already mentioned idea of having a specialized nurse, which already exists in other health-care settings. A review, which focuses on the effectiveness of specialized nurses in long-term care,^24^ has shown that they are associated with improvements in several measures of health status and behaviors of elderly people. Consequently, such an approach would provide targeted and person-centered services to vulnerable or impaired elderly. As frailty is a dynamic concept,^25^ elderly health could be improved in a sustainable way.

Third, the need for structure in daily life incorporating activities was deemed as very important as it supports committed and regular participation of planned activities that creates a feeling of success. A study analyzing the relationship between daily living, quality of life, social support and depression levels of elderly individuals in Turkey^26^ has shown that living with a spouse or family member positively influences the level of activities in daily life. This highlights the close connection with the need for social relationships, which will be discussed in detail below.

Lastly, the fourth need highlights the importance of the sense of community and belonging. Our results have shown that activities are more likely to be undertaken if they are conducted with the same people. Importantly, not only relatives but also the local community have an influence on the activities of the elderly. Future programs and activities should therefore take place repeatedly within the same local groups. This would have a positive effect on self-efficacy which is strongly influenced by the social environment.^10^ Social persuasion and seeing persons in similar situations succeed have a positive effect on a person’s belief that they can cope with a situation and therefore has a positive effect on self-efficacy. This implies that these programs and activities should be conducted within groups of people with similar risk factors and activity levels. Earlier studies have also confirmed that individuals with higher self-efficacy generally benefit more from health education programs.^27^ Moreover, their quality of life increases and the compliance for health-related self-care activities is affected positively.^27^ A review including 6 studies has highlighted that a lack of social contact and accompanying social isolation have a negative effect on health,^20^ where the elderly are more likely to be affected than the rest of the population. This was also corroborated by the participants in this study, who mentioned the importance of establishing a social network, the sense of belonging, as well as the fear of being alone. This need is also supported by previous studies which have shown the protective effects of social networks on morbidity and mortality.^13,22^ Consequently, elderly people who are embedded in social networks have been found to not only enjoy better physical, but also mental health.^28^ Our study’s findings have also shown that people change their behavior and step outside their comfort zone to build a local network to meet these needs, which is often experienced as not easy. Considering a social network as an essential part of healthy aging, it is important to support the population within this establishing process. The already mentioned circle of care concept^14^ is a model that could support create conditions that allow elderly people to build and maintain social contacts more easily. As it strengthens the whole local social environment and is not limited to caring family members or outpatient care providers. This makes it possible to see the interfaces between the different dimensions and how the focal points of care can be coordinated in a more community centered way.^14^

### Limitations

Three major limitations need to be addressed. First, the generalizability of the results is limited as the sample is homogeneous and not representative given a high share of the participants were female, and socioeconomic factors within the group were similar with all participants living in the same city. However, the sample still meets the relevant characteristics of the population of interest in Switzerland. Second, the double translation of the data from Swiss-German to German during the transcription process and then from German to English might have affected the authenticity of the collected data due to translation difficulties. Third, the entire analysis was performed by the first author, which may influence the reliability of the results.

## Conclusion

To create adequate services that support elderly people in their health-related needs, it is important to understand and include their perspective and experiences. Through interviews, 4 main categories have been identified as important for their health-related needs. The general living conditions and social environment are considered extremely important as these strongly influence the way a person deals with their own health. Therefore, focusing more on community-based approaches like the circle of care is a promising way of improving healthy aging. This study also implies that specialized nurses, who conduct home visits using a frailty assessment that identifies health risks and disabilities, may be an adequate approach to provide targeted support to elderly people, which may include educational and counseling interventions, as well as activities. These could be offered and conducted within local groups with similar risk factors.

This study therefore suggests the implementation of alternative community-centered models of health care services and programs, based on the experienced needs of the elderly population. In order to achieve this, further research is necessary to gain a deeper understanding of these needs and the impact of such approaches.
